# Venetoclax treatment of patients with relapsed T-cell prolymphocytic leukemia

**DOI:** 10.1038/s41408-021-00443-1

**Published:** 2021-03-02

**Authors:** Paul J. Hampel, Sameer A. Parikh, Timothy G. Call, Mithun V. Shah, N. Nora Bennani, Aref Al-Kali, Kari G. Rabe, Yucai Wang, Eli Muchtar, Jose F. Leis, Saad S. Kenderian, Amber B. Koehler, Susan M. Schwager, Susan L. Slager, Neil E. Kay, Curtis A. Hanson, Daniel L. Van Dyke, Min Shi, Wei Ding

**Affiliations:** 1 Division of Hematology, Department of Medicine, Mayo Clinic, Rochester, MN USA; 2 Division of Biomedical Statistics & Informatics, Department of Health Sciences Research, Mayo Clinic, Rochester, MN USA; 3 Department of Hematology and Oncology, Mayo Clinic, Phoenix, AZ USA; 4 Department of Laboratory Medicine and Pathology, Mayo Clinic, Rochester, MN USA

**Keywords:** Leukaemia, Molecularly targeted therapy, Leukaemia

**Dear Editor**,

T-cell prolymphocytic leukemia (T-PLL) is a rare, aggressive malignancy of post-thymic mature T cells. Historically poor outcomes with conventional chemotherapy preceded the establishment of the current standard frontline treatment approach with intravenous administration of the anti-CD52 antibody alemtuzumab^1^. Despite initial high response rates with alemtuzumab, relapse is inevitable without a consolidative hematopoietic stem cell transplant (HSCT)^2^. Yet, most patients are ineligible for HSCT, either due to age, comorbidities, or lack of a durable response to initial therapy, and relapsed/refractory disease carries a dismal prognosis^3^. Venetoclax, an oral inhibitor of the anti-apoptotic protein BCL-2, has demonstrated impressive efficacy in the management of multiple hematologic malignancies. Strong responses to venetoclax on ex vivo drug sensitivity screens suggest that it may have a role in the treatment of T-PLL patients^4,5^. Prior studies have reported three patients who achieved partial remission (PR) with venetoclax monotherapy^5,6^ and a more durable (10 months) complete response with combination venetoclax and pentostatin^7^. Recently, additional case reports have also suggested superior responses when venetoclax was administered in a combination approach^8–10^. Herein, we report outcomes of patients with relapsed/refractory T-PLL treated with venetoclax at our institution. Using an institutional clinical database of patients with T-PLL seen in the Division of Hematology at Mayo Clinic, Rochester, MN, we identified 9 T-PLL patients who received venetoclax between 8/2017 and 5/2020. Diagnostic criteria and response definitions were utilized as per the T-PLL International Study Group^11^.

The median age was 63 years (range 49–75); individual patient characteristics are detailed in Table 1 (patients referenced by # in Table from here on forward). Two patients (#1 and #8) had *JAK3* mutations and patient #8 also had overexpression of BCL2 on RNA sequencing (additional sequencing, karyotype, and laboratory details in Supplemental Material). The median number of prior lines of therapy was 3 (range 1–4), including alemtuzumab in 8 of 9 patients, and two patients had undergone prior HSCT after achieving a complete remission. The median time from T-PLL diagnosis to start of venetoclax was 12 months (range 3–22 months). Eight out of 9 patients had active disease prior to venetoclax initiation; 1 patient (#2) started venetoclax as maintenance following PR from prior treatment. Active disease defining features present included: disease-related constitutional symptoms (*n* = 7), cytopenias (*n* = 7), nodal/splenic disease (*n* = 6), increasing lymphocytosis (*n* = 7), and extranodal involvement (*n* = 6; cutaneous [*n* = 3], effusions [*n* = 5]).

**Table 1:** Clinical and pathologic features in individual patient disease courses.

	Patient 1	Patient 2^a^	Patient 3	Patient 4	Patient 5	Patient 6	Patient 7	Patient 8	Patient 9
Demographics, comorbidities, and disease characteristics									
Age (y) at Ven start and gender	62 M	49 M	61 F	75 F	63 F	63 F	73 M	71 F	59 F
CIRS score	6	6	2	6	10	4	5	3	13
CIRS organ systems	Vasc, Resp, UGI, LGI	Vasc, Resp, UGI	Resp, GU	Heart, Vasc, UGI	Heart, Vasc, Resp, Renal, Psych	Vasc, UGI	Vasc, UGI, ENT	Endo, MSK, Psych	Heart, Vasc, Resp, UGI, Endo, Psych
Flow cytometry	CD4−/CD8+	CD4+/CD8−	CD4+/CD8+	CD4+/CD8−	CD4−/CD8−^b^	CD4+/CD8−	CD4+/CD8−	CD4+/CD8+	CD4+/CD8−
Complex karyotype	No	NA	Yes	Yes	NA	Yes	NA	Yes	No
FISH (% nuclei)	*TRAD* (68%), *TCL1A*x3 (68.5%)	*TCL1A* (88.5%)	*TCL1A* (97%)	*TCL1A* (52.5%), *TCLA1A*x3 (22.5%), *TRAD* (64%), *TRAD*x1 (22%)	NA	*TCL1A* (39.5%)	*TCL1A* (60%)	*TRAD* (74%), trisomy 8 (56%)	*TCL1* (44%)
* JAK* mutations	*JAK3* (pA573V), VAF 35%	NA	NA	NA	NA	NA	NA	*JAK3* (pM511I), VAF 42%; *JAK3* (pA572V), VAF 34%	NA
Prior lines of therapy	alemtuz, HDMTX + IT cytara, alemtuz + pentostatin, Flu + Mel + TBI + MUD HSCT	alemtuz, benda, benda	alemtuz + CTX + Flu + mitox, alemtuz, alemtuz + pentostatin, benda + vorinostat	alemtuz, benda, romidepsin	CHOP, gemcitabine + oxaliplatin, pralatrexate	CHOP, alemtuz,^c^ romidepsin	alemtuz, BEAM + auto-HSCT	alemtuz	alemtuz
Clinical presentation at venetoclax start									
Months from diagnosis to Ven start	16	22	8	12	19	3	13	9	10
Fatigue or B symptoms	None	None	Fatigue (PS 3)	Fatigue (PS 3)	Fatigue (PS 3) and B sx	Fatigue (PS 2)	Fatigue (PS 2)	B sx	Fatigue (PS 2)
Extranodal sites	Cutaneous, ascites	None	None	None	Ascites	Cutaneous	Ascites	Pleural effusion	Cutaneous, ascites
Spleen size (cm)	23	13.5	15.4	15	28	14.5	18	16	16
Largest lymph node size (cm)	2.2	<1	<1	4.7	2.2	3.2	3.5	2.2	<1
WBC (×10^9^/L)	10.2	8.7	480.4	235.5	59.8	17.3	63.1	93.8	202.4
LDH (U/L)	377	191	4236	4205	228	1659	2301	862	1947
Hgb (g/dL)	9.4	14.1	6.3	6.9	9.5	12.3	6.7	9.3	10.0
Platelets (×10^9^)	31	96	10	41	80	190	47	84	73
Bone marrow involvement (%)	90	NA	90	NA	30	10	60	NA	60
Venetoclax treatment details and outcomes									
Max Ven dose (mg)	50	200	200^d, e^	100^d, e^	800^d^	800^d^	400	800^d^	800^d^
Ven duration (days)	25	42	4	4	171	30	177	201	101
Concomitant steroids at Ven start	None	None	MP 2 g × 2d, 1 g × 3d	MP 500 mg × 1d, 1 g × 1d, 2 g × 1d	None	MP 250 mg × 5d	MP 1 g × 2d, 500 mg × 1d	MP 2 g × 3d	MP 1 g × 2d
Overlapping benda	90 mg/m^2^ 1 cycle	None	None	None	None	100 mg/m^2^ 1 cycle	70 mg/m^2^ 4 cycles	60 mg/m^2^ 1 cycle	50–70 mg/m^2^ 3 cycles
Ven best response	PD	PD	PD	PD	SD	PR	PR	PR	PR
Subsequent therapies	None	benda	brentuximab vedotin	None	None	None	alemtuz + cladribine + vorinostat	None	alemtuz + pentostatin
Survival from Ven start (days)	30	53	6	4	464	34	200	201	120

*Alemtuz* alemtuzumab, *auto* autologous, *BEAM* carmustine, etoposide, cytarabine, melphalan; benda: bendamustine, *CIRS* Cumulative Illness Rating-Scale, *CHOP* cyclophosphamide, doxorubicin, vincristine, prednisone, *CTX* cyclophosphamide; cytara: cytarabine, *d* days, *Dx* diagnosis, *FISH* fluorescence in situ hybridization, *Flu* fludarabine, *HDMTX* high-dose methotrexate, *Hgb* hemoglobin, *HSCT* hematopoietic stem cell transplantation, *g* grams, *LN* largest lymph node by imaging, *Mel* melphalan, *mg* milligrams, *mitox* mitoxantrone, *MP* methylprednisolone, *MUD* matched unrelated donor allogeneic, *ND* not done, *PD* progressive disease, *PR* partial remission, *PS* ECOG performance status, *Pt* patient, *S* sex, *SD* stable disease, *TBI* total body irradiation, *TCL1A* 14q32, *TRAD* 14q11.2, *VAF* variant allele frequency, *Ven* venetoclax, *y* years.

^a^Patient was in PR following prior line 6 cycles bendamustine; all other patients actively progressing at Ven start.

^b^Although CD4−/CD8− is a rare immunophenotype in T-PLL, the additional immunophenotype (CD1a−, CD16−, CD56−, and CD57−) and morphological (marked nuclear irregularity and distinct nucleoli) findings in combination with the clinical presentation were considered most consistent with a diagnosis of T-PLL.

^c^Alemtuzumab subcutaneous. All of the other patients treated with alemtuzumab received intravenous alemtuzumab.

^d^Underwent venetoclax rapid dose escalation.

^e^Patient receiving concurrent CYP3A4 inhibitor (pt #3: posaconazole; pt#4 diltiazem).

Three patients initiated venetoclax with a weekly ramp-up as per the package insert chronic lymphocytic leukemia (CLL) schedule^12^; two of them received concomitant bendamustine. Six patients underwent rapid dose escalation (detailed in Supplemental Material); three of them received concomitant bendamustine. Altogether, bendamustine was given with venetoclax to 5/6 patients who were bendamustine-naive. The target maximum dose of venetoclax (800 mg [*n* = 4]; 400 mg [*n* = 1]) was reached in 5 patients at a median of 12 days (range 7–40 days). The other four patients had disease progression during the dose ramp-up.

The disease control rate was 56%; best response was PR in 4 (44%) patients (Fig. 1B–E) and stable disease (SD) in 1 (11%) patient. The one patient with SD received venetoclax monotherapy; however, the overall response rate (ORR) among patients who received the combination of venetoclax and bendamustine was 80% (4/5 patients). Both patients who had received only 1 prior line of therapy (alemtuzumab) responded (100% ORR). This includes patient #8, who met all criteria for complete remission but did not have a confirmatory bone marrow biopsy. Cutaneous disease improved in 2/3 patients (both with PR as best response), and effusions improved in 2/5 patients (1 PR and 1 SD as best response).

**Fig. 1:**
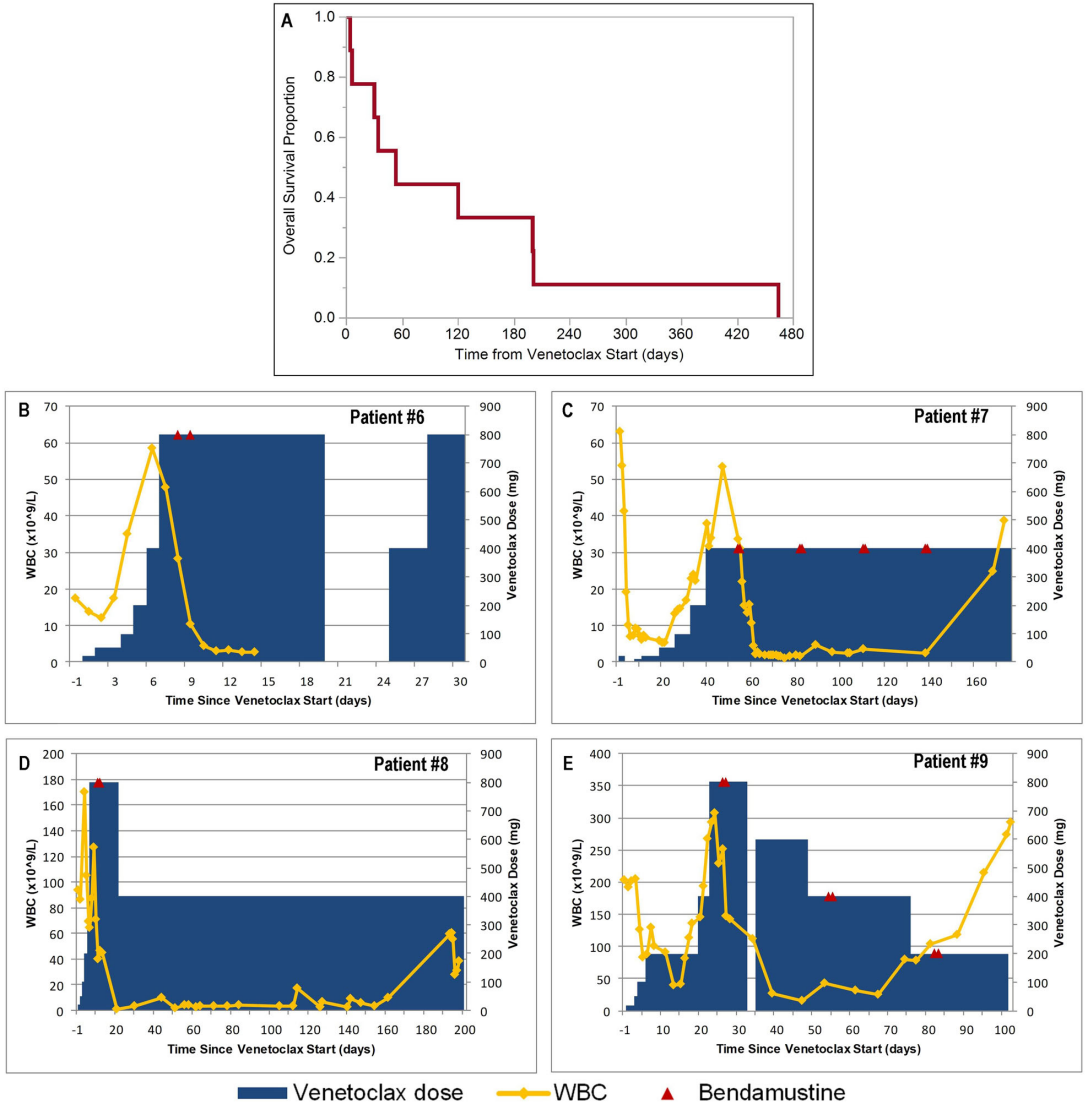
Survival outcomes and clinical courses with venetoclax treatment. Overall survival of all patients from the start of venetoclax treatment (**A**) White blood cells (WBC) during venetoclax and bendamustine dosing among patients who experienced clinical response (*bottom panels*): Patient #6 (**B**), Patient #7 (**C**), Patient #8 (**D**), Patient #9 (**E**).

Variable sensitivity to venetoclax during the first few doses was observed. Small initial doses produced a dramatic and immediate decrease in lymphocytes in two patients (#7 and #9). A subsequent rise in lymphocytes while still undergoing dose escalation occurred in both patients, but ultimately PR was achieved at target doses (Fig. 1). The other two patients with PR had increasing leukocytosis during the dose ramp-up without a preceding decrease prior to reaching maximum dosing. Still, a >50% decrease in lymphocytes was observed either before reaching their target venetoclax dose or within 5 days afterwards in all four patients with eventual T-PLL-ISG responses. Further exemplifying the capacity for proliferative disease at treatment start, two patients (#3 and #4) suffered fatal disease progression within 1 week of venetoclax initiation despite rapid dose ramp-up and high-dose corticosteroids delivered with temporizing intent. Significant leukocytosis (480 and 235 × 10^9^/L) and elevated lactate dehydrogenase (>4200 U/L) at venetoclax start were common features among these two patients. Another heavily pre-treated patient (#1) died with progressive disease during standard dose escalation despite concomitant bendamustine. Predictive biomarkers for sensitivity to venetoclax are not yet known, but these findings suggest venetoclax may be insufficiently active in unselected patients with high disease burden. Median duration of treatment for all patients was 42 days (range 4–201 days). All patients ultimately died during follow-up with a median overall survival of 53 days (range 4–464 days); Fig. 1A.

Using Common Terminology Criteria for Adverse Events v5.0.^13^, all patients experienced at least one adverse event, and 8/9 patients had a grade ≥3 toxicity, most commonly edema (*n* = 7) and neutropenia (*n* = 6). Five patients required dose interruptions due to neutropenia (*n* = 3), clinical tumor lysis syndrome (*n* = 1), and edema (*n* = 1). Infections while on therapy included grade 3 pneumonia, grade 3 cellulitis, and grade 2 CMV reactivation. Three patients had dose reductions, all from 800 mg, due to hematologic toxicity (*n* = 2) and nausea (*n* = 1).

Due to the rarity of T-PLL, treatment guidance relies heavily on retrospective analyses and small prospective studies, particularly in the relapsed/refractory setting. The benefit of single agent venetoclax in this cohort was limited to a single observation of stable disease as best response. Treatment with venetoclax in combination with bendamustine showed modest efficacy, achieving an encouraging 80% ORR in bendamustine-naive patients. However, survival remained short even among these patients (range 34–201 days).

Treatment with combination alemtuzumab and cladribine (with or without an HDAC-inhibitor) was very effective (100% ORR) in a retrospective cohort including 6 patients with relapsed disease and prior alemtuzumab exposure^14^. However, this regimen carries significant infectious risk and hematologic toxicity which may preclude routine use. The literature regarding non-alemtuzumab-based approaches is limited. A larger retrospective study showed pentostatin led to a response in 11/24 (46%) patients with previously treated T-PLL^15^. Bendamustine alone achieved a 43% ORR in seven patients with relapsed/refractory T-PLL who had only received prior alemtuzumab in a retrospective study^16^. In that study, 4 patients progressed after 2 cycles of bendamustine, 1 patient had an ongoing response after 3 cycles, and 2 patients had durable responses of 13 and 27+ months after 6 cycles^16^. Two patients (#8 and #9) in our study had similarly only received frontline alemtuzumab; each patient was treated with combination venetoclax and bendamustine (1 cycle and 3 cycles), and both responded (durations of approximately 7 and 3 months).

Herbaux et al. suggested a higher response rate may be associated with high-dose bendamustine (120 mg/m^2^) in monotherapy treatment^16^, but severe hematologic toxicities are frequent with this dosing. Combination with venetoclax is prohibitive to higher doses of bendamustine due to cytopenias. However, our findings suggest the addition of venetoclax may allow for fewer cycles of bendamustine and avoid the need for these higher doses, potentially improving tolerability in doing so. Still, the frequent neutropenia observed in the current study highlights the need for combination approaches with less overlapping toxicity. Similarly, the encouraging complete response reported with pentostatin and venetoclax was also complicated by hematologic toxicity^7^.

Ibrutinib and venetoclax are a pairing which has shown synergy in some^9^, but not all^6^, laboratory investigations with T-PLL samples and with reported tolerability in patients with CLL^17^. Two clinical responses^9^ and a period of stable disease halting exponential proliferation in another case^10^ have been reported also, and a clinical trial (NCT03873493) is underway to further evaluate this combination in patients with T-PLL. Utilizing a multi-agent regimen targeting key pathways in T-PLL, a remarkable response with venetoclax plus alemtuzumab, cladribine, and vorinostat was described in a patient with very active disease and who previously had progressed during venetoclax monotherapy ramp-up^8^. Collectively, our findings and these reports emphasize the optimal role for venetoclax is likely as part of a combination regimen.

Despite advances in the molecular characterization of T-PLL identifying the importance of the JAK/STAT pathway and epigenetic modifiers^18,19^, the therapeutic impact of this knowledge has yet to be realized. While functional drug screens obviate some of this complexity, the transient responses observed with venetoclax monotherapy reveal their limitations. Our current study represents the largest cohort of patients with T-PLL treated with venetoclax reported, to the best of our knowledge. No clear pattern of responses was observed across clinically available flow cytometry and cytogenetic data. BCL2 overexpression, which has correlated with venetoclax activity in T-PLL samples^5^, was found in the patient (#8) who achieved the best response among this cohort; however, the retrospective nature of this study and lack of research blood samples for additional testing limit any conclusions regarding molecular correlations. Future efforts to identify predictive biomarkers for venetoclax, as well as optimal combination strategies, are required. As it stands yet, treatment of patients with relapsed/refractory T-PLL remains a significant unmet need.

## Supplementary information

Supplemental Material
