# Supplementary material for: Venetoclax treatment of patients with relapsed T-cell prolymphocytic leukemia
Source: Blood Cancer J. 2021 Mar 2;11(3):47. doi: 10.1038/s41408-021-00443-1 (PMC7925672; doi:10.1038/s41408-021-00443-1)
Supplement: Supplementary file 1 — Supplemental Material [file 41408_2021_443_MOESM1_ESM.pdf]

SUPPLEMENTAL INFORMATION:

**Rapid dose escalation administration:**

Patient #3: 20 mg x 1 day, 50 mg x 1 day, 100 mg x 1 day, 200 mg x 1 day (day 4). The patient discontinued after these 4 days of treatment amidst progressive leukocytosis.

Patient #4: 20 mg x 1 day, 50 mg x 2 days, 100 mg x 1 day (day 4). The patient had progressive worsening of disease throughout these four days and passed away on day 4 after transitioning to comfort measures only.

Patient #5: 20 mg x 1 day, 50 mg x 2 days, 100 mg x 4 days, 200 mg x 2 days, 400 mg x 2 days, and reaching 800 mg on day 12.

Patient #6: 20 mg x 1 day, 50 mg x 2 days, 100 mg x 1 day, 200 mg x 1 day, 400 mg x 1 day, and reaching 800 mg on day 7.

Patient #8: 20 mg x 1 day, 50 mg x 1 day, 100 mg x 1 day, 200 mg x 2 days, 400 mg x 1 day, and reaching 800 mg on day 7.

Patient #9: 20 mg x 1 day, 50 mg x 2 days, 100 mg x 2 days, 200 mg x 14 days, 400 mg x 3 days, and reaching 800 mg on day 23.

**Complex karyotype details**:

Patient #3: 43-45,XX,add(1)(q32),+3,del(3)(q23),add(3)(q27),-5,-6,-7,der(7)add(7)(p13)add(7)(q32),+8,der(8)t(6;8)(p11.2;p21)x2,i(8)(q10),-9,add(9)(p13),del(10)(q24),del(11)(q23),add(12)(p11.2),inv(12)(p13q24.3),-13,add(13)(p11.2),-14,-15,-16,add(17)(p11.2),-18,add(18)(q21),-20, add(20)(p11.2),-21,add(21)(q22),-22,+3-6mar[cp15]

Patient #4: (43-46,XX,i(8)(q10),-13,-14,der(14)inv(14)(q11.2q32)add(14)(p 11.2),-22,+0-4mar[cp13]/46,XX[7])

Patient #6: 45,X, der(X)t(X;9)(q28;q22), add(3)(q29), add(6)(q23), t(6;18)p21;q23), t(7;10)(13;q22), +der(7)t(7;10), +8, der(8)t(8;8)(p21;q22)x2, -9,-10, add(11)(q21), add(12)(p13), del(12)(p12), -14[14]/46,idem,+r[6]

Patient #8: 47,t(X;14)(q28;q11.2)x2,+8,der(8;22)(q10;q10),-11,add(11)(p1 1.2), +14, +mar[1]/46,XX[9])

**Tempus commercial sequencing details**:

Patient #1: *JAK3* (pA573V) mutation with variant allele frequency 34.6%. Tumor Mutational Burden was 5.3 m/MB ($86^{th}$ percentile). Tempus xO 1714 Genes panel with Pipeline Version 1.3.5.

Patient #8: *JAK3* (pM511I) mutation with variant allele frequency 41.9%, *JAK3* (pA572V) with variant allele frequency 33.5%, *NBN* (pK219fs) with variant allele frequency 60%, and *FBXW7* (pA252fs) with variant allele frequency 38%. RNA sequencing also performed by Tempus

showed overexpressed BCL2, BRAF, CREBBP, MAPK1, MAP2K1, MYC. Tempus xO 1714 Genes panel with Pipeline Version 1.6.3.

**MatePair targeted rearrangement details**:

Methods: Library preparation was performed using the Nextera mate pair library preparation kit. Samples were sequenced on the Illumina HiSeq 2500, and sequencing reads were mapped to the GRCh38 genome build.

Patient #8: Mate pair sequencing detected a t(X;14)(q28;q11.2). This rearrangement involves the TRA locus (14q11.2) (BMD_TRA_locus) and intron 1 of the MTCP1 gene (Xq28) (NM_001018025). Sanger sequencing confirmed this gene rearrangement.

**FISH locus and probes**: 14q11.2 (5'TRAD, 3'TRAD), break-apart probe strategy; 14q32.1 (5'TCL1A, 3'TCL1A), break-apart probe strategy; 8q24 (*MYC*), copy number strategy; 7q31 (D7S486), copy number strategy

**T-cell receptor gene rearrangement method summary**: A PCR-based assay was performed on extracted DNA using primers that bind the gamma and beta chain genes.
